# Supplementary material for: Rapid evolution of dispersal ability makes biological invasions faster and more variable
Source: Nat Commun. 2017 Jan 27;8:14315. doi: 10.1038/ncomms14315 (PMC5290149; doi:10.1038/ncomms14315)
Supplement: Supplementary Information — Supplementary Figures 1-8 and Supplementary Tables 1-8 [file ncomms14315-s1.pdf]

Supplementary Figures

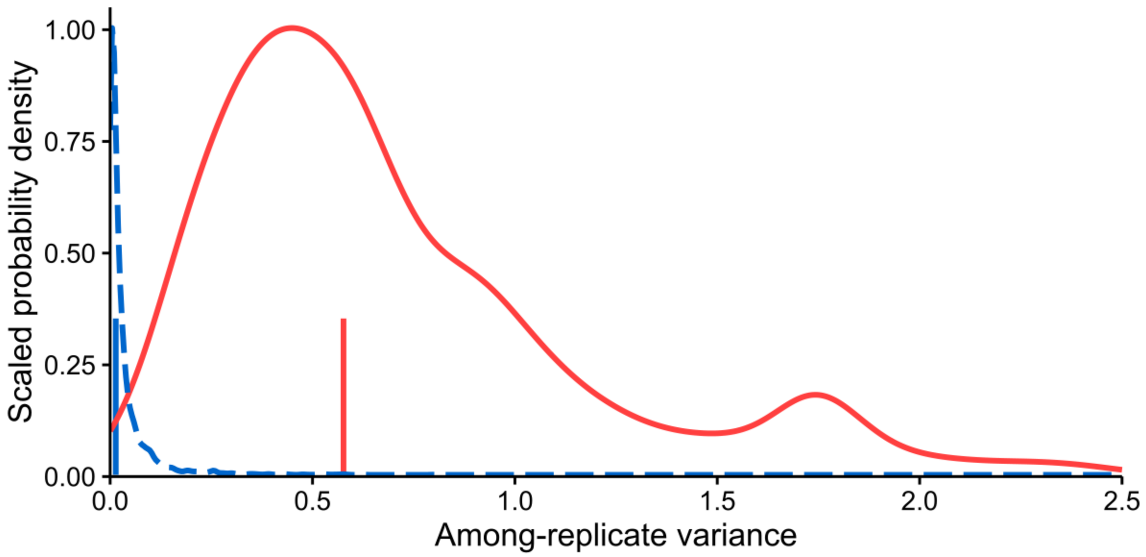

Supplementary Figure 1 | Comparison of among-replicate variance in invasion dynamics

Scaled posterior probability densities for among-replicate variances in invasion speed (nine replicates per treatment). Spatially sorted replicate populations (red solid line) have higher among-replicate variance in invasion speed than shuffled replicate populations (blue dashed line) (model selection results in Supplementary Table 2). Vertical red and blue lines show median estimates for spatially sorted and shuffled replicates, respectively.

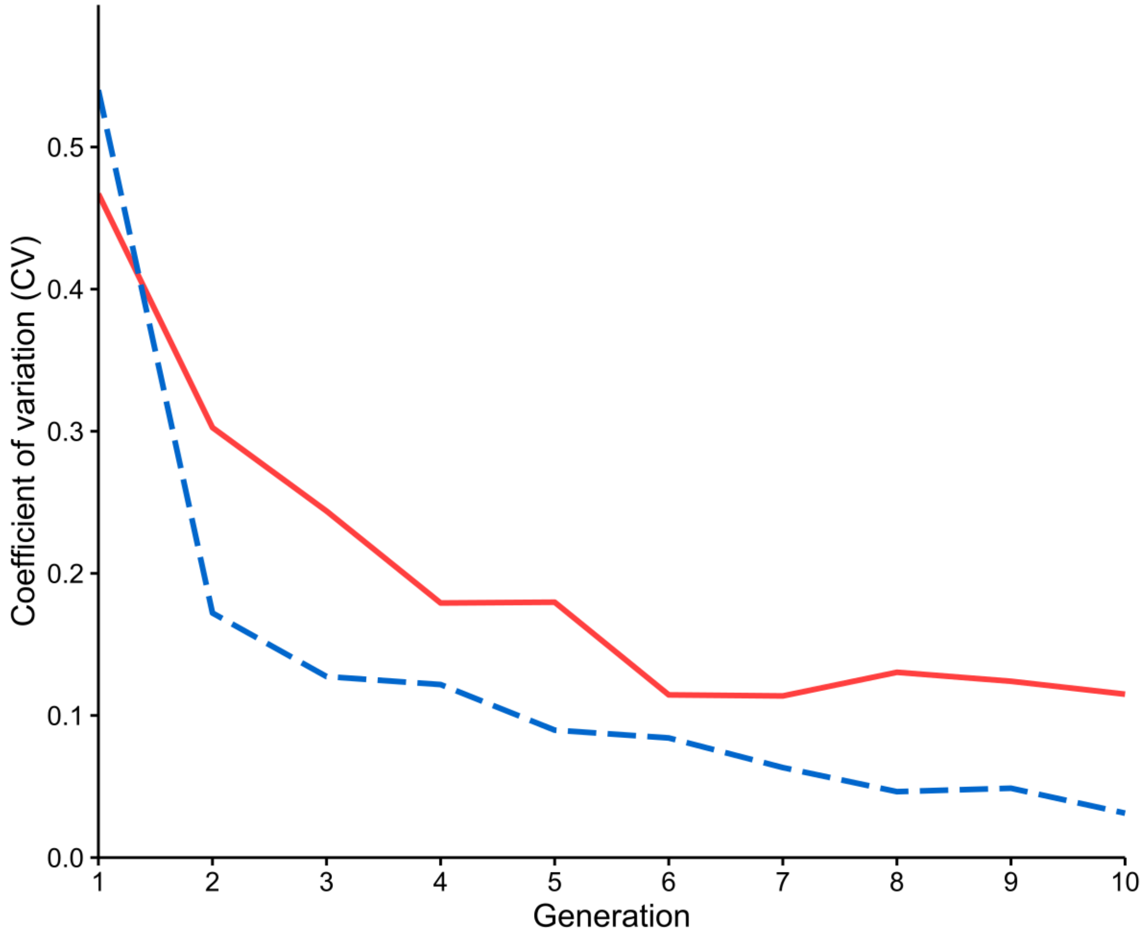

Supplementary Figure 2 | Comparison of CV in invasion extent between treatments

Lines show estimated coefficient of variation (CV) in invasion extent for spatially sorted (red solid line) and shuffled (blue dashed line) invasion treatments (nine replicates each). Since mean invasion extent within treatment was low for the first generation relative to the variance in invasion extent (Fig. 2), both treatments had initially high CVs that decreased over time. However, the CV for sorted invasions decreased at a slower rate relative to the CV for shuffled invasions, resulting in an overall higher CV for sorted invasions at the end of the experiment.

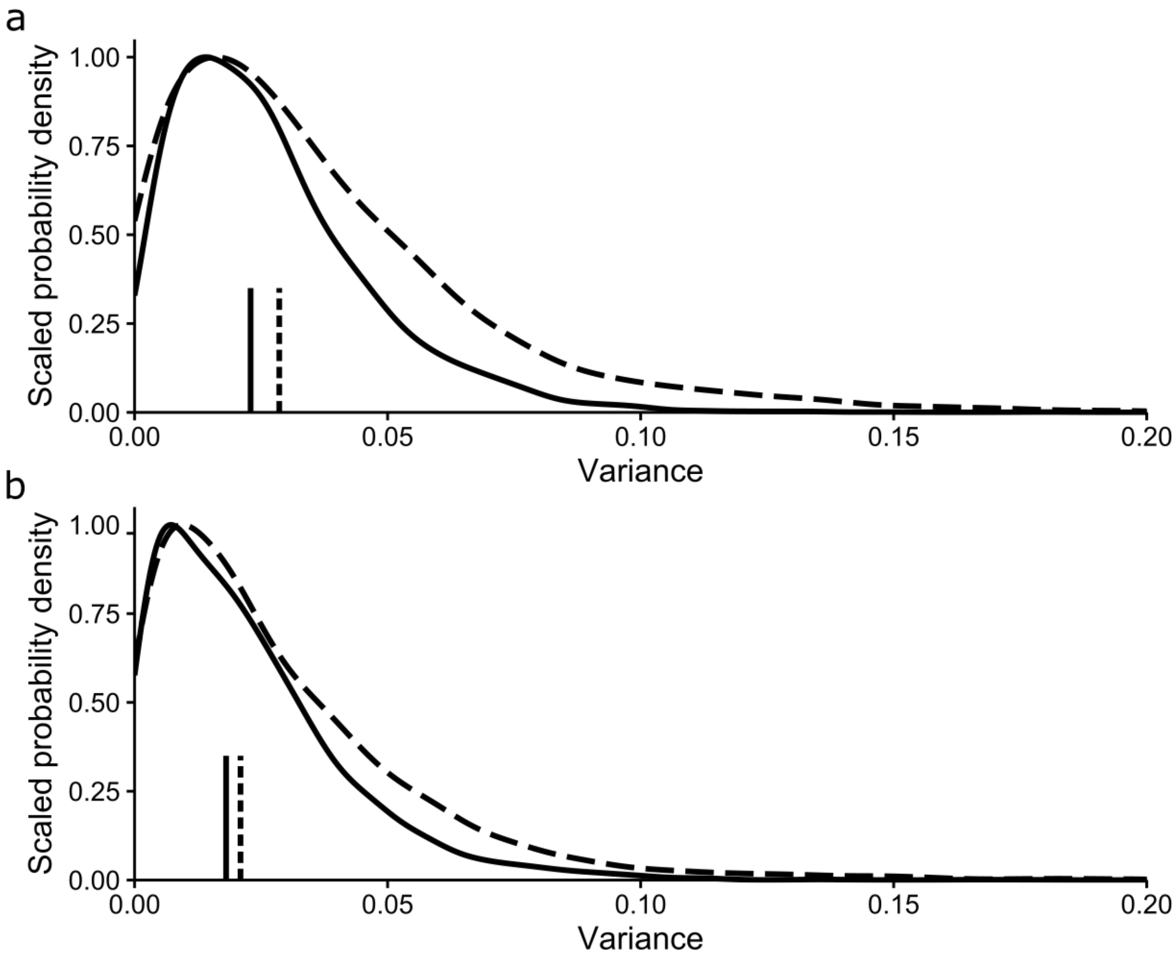

Supplementary Figure 3 | Additive genetic variance in dispersal ability

Scaled posterior probability densities for dam variance (solid lines) and sire variance (dashed lines) in dispersal ability (Methods). Vertical lines show median estimates. Females (N = 537) **(a)** and males (N = 513) **(b)** were analyzed separately. Models that accounted for sire variance in dispersal ability provided a better fit to dispersal data from a nested paternal half-sib breeding experiment than models which did not account for sire variance (model selection results in Supplementary Table 3), suggesting that dispersal is a heritable trait in *C. maculatus*.

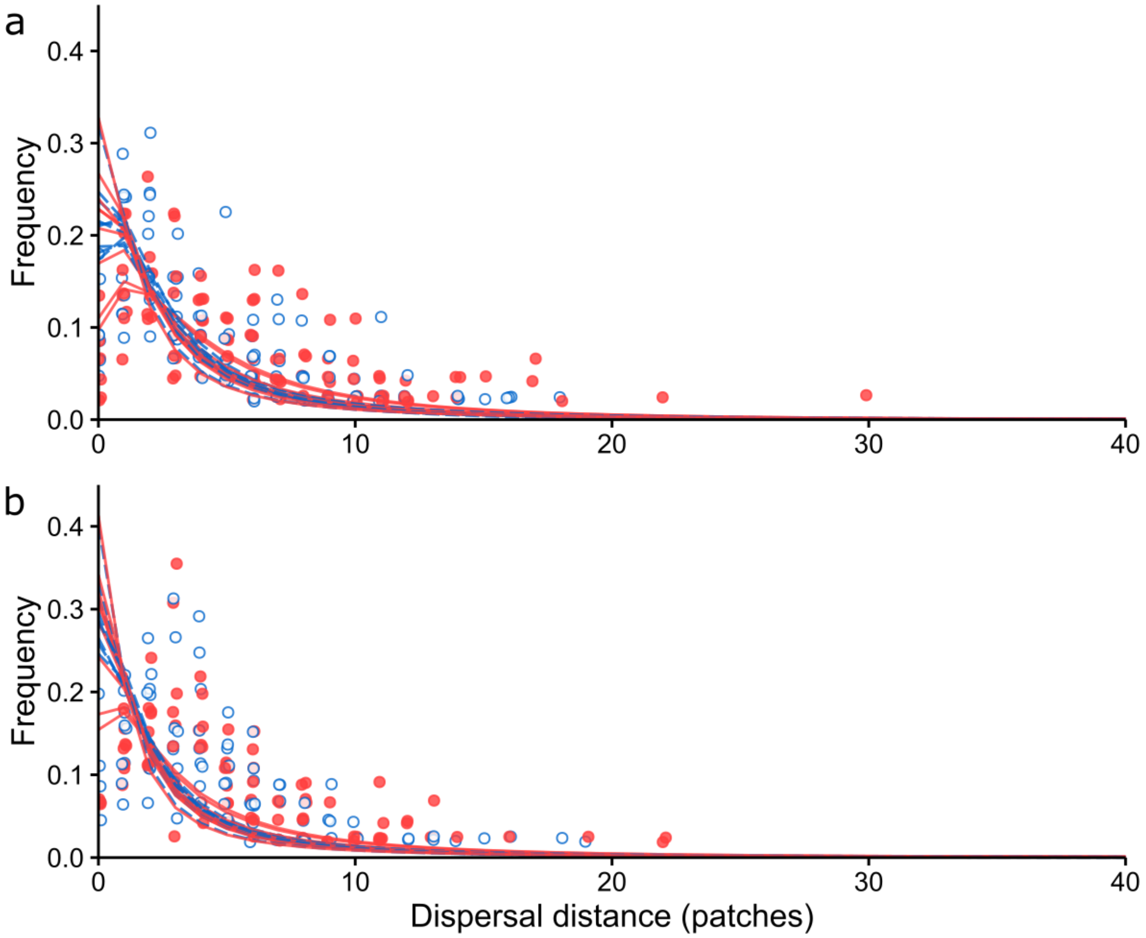

Supplementary Figure 4 | Comparison of female post-invasion dispersal kernels

Best-fit PIG dispersal kernels for females in spatially sorted (red solid lines, closed circles) and shuffled (blue dashed lines, open circles) populations (nine replicates each), following two generations in a common environment. Panels show best-fit kernels from **(a)** the first generation (*N* = 1620) and **(b)** the second generation (*N* = 1620) (model selection results in Supplementary Table 4). Lines show best-fit kernels for each population; points show raw data.

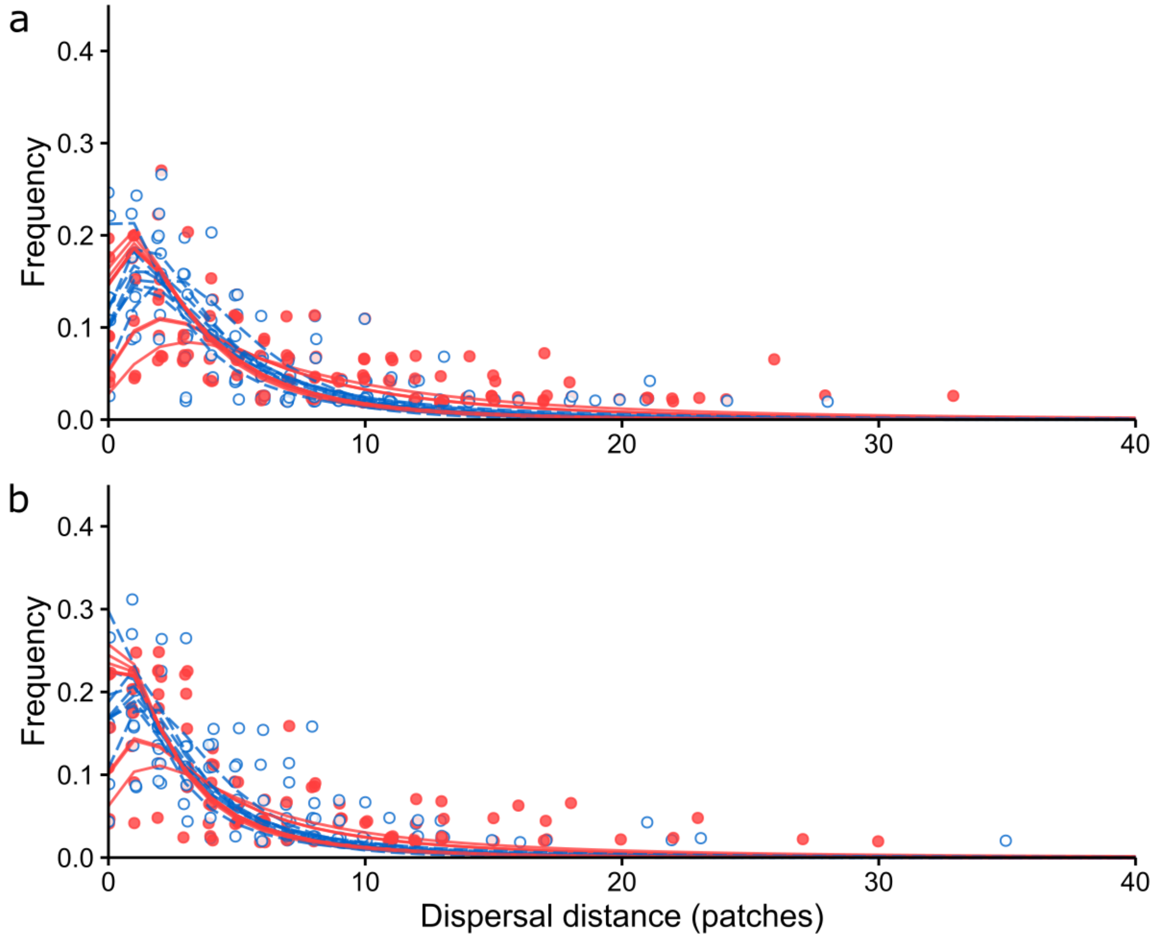

Supplementary Figure 5 | Comparison of male post-invasion dispersal kernels

Best-fit PIG dispersal kernels for males in spatially sorted (red solid lines, closed circles) and shuffled (blue dashed lines, open circles) populations (nine replicates each), following two generations in a common environment. Panels show best fit kernels from **(a)** the first generation (*N* = 1620) and **(b)** the second generation (*N* = 1620) (model selection results in Supplementary Table 5). Lines show best-fit kernels for each population; points show raw data.

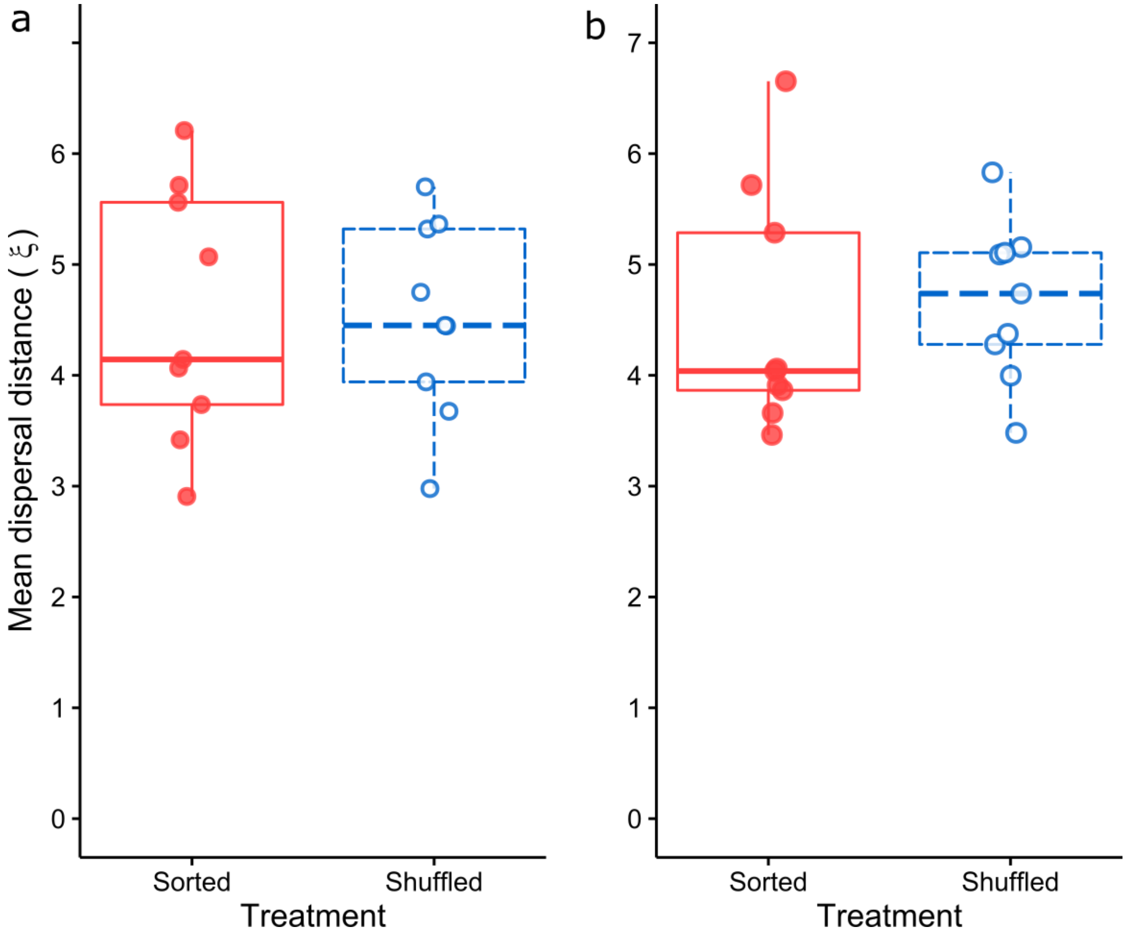

Supplementary Figure 6 | Mean pre-invasion dispersal distance

Tukey boxplots showing estimates for mean dispersal distance (ξ) at the start of the experiment (Methods). Distance, in number of patches, is shown for spatially sorted (red solid boxes, closed circles) and shuffled (blue dashed boxes, open circles) populations (nine replicates each). Points show mean estimates, and are jittered along the x-axis to reduce overlap. Females **(a)** and males **(b)** were analyzed separately (model selection results in Supplementary Table 6).

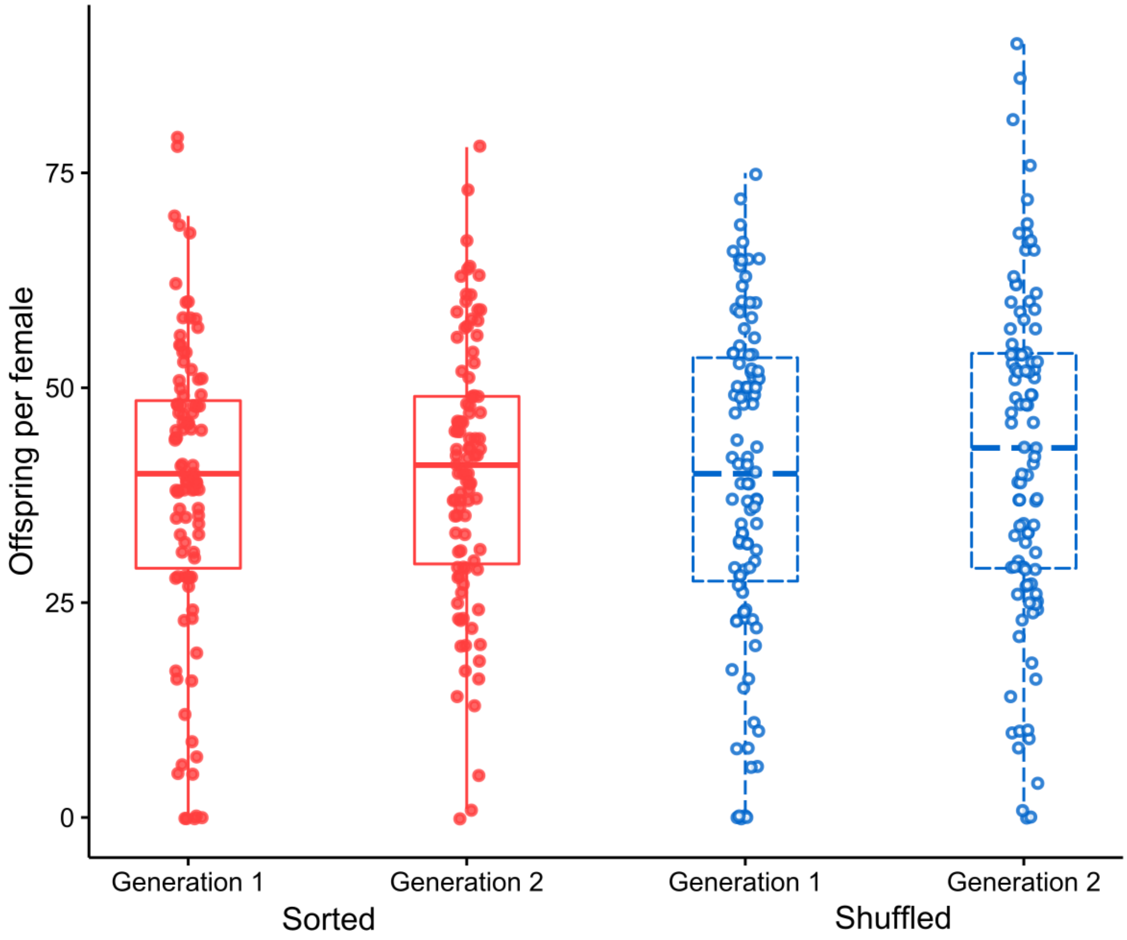

Supplementary Figure 7 | Comparison of post-invasion reproductive rates

Tukey boxplots showing the number of offspring per female in spatially sorted (red solid boxes, closed circles) and shuffled (blue dashed boxes, open circles) replicates, following two generations in a common environment (nine replicate populations for each treatment, 11 females per replicate per common garden generation, $N = 396$; model selection results in Supplementary Table 7). Points show raw data, and are jittered along the x-axis to reduce overlap.

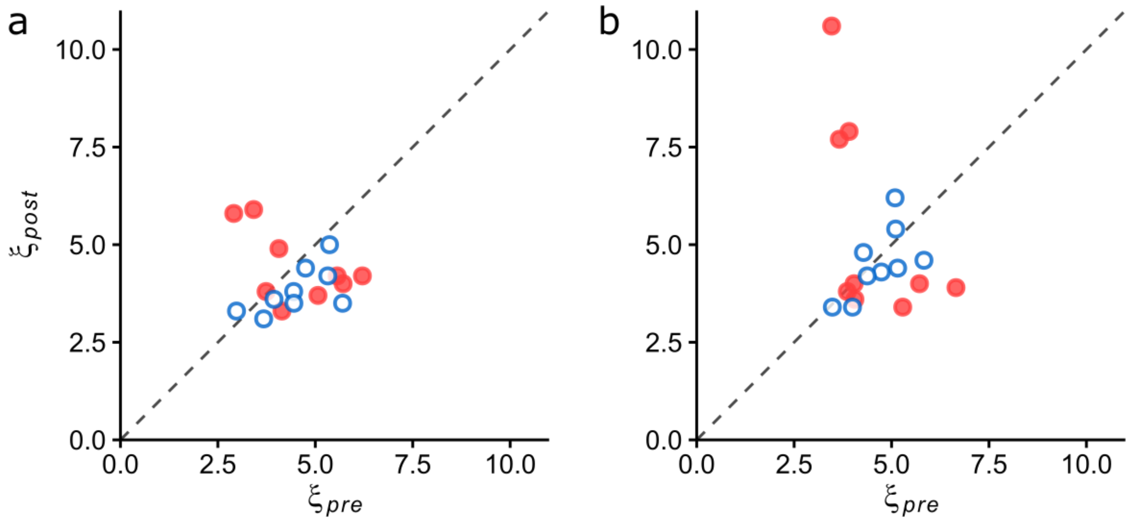

Supplementary Figure 8 | Comparison of pre- and post-invasion mean dispersal distance

Scatterplots of estimated mean dispersal distance for pre-invasion (ξ_{pre}) and post-invasion (ξ_{post}) dispersal kernels (Methods). Points show mean dispersal distances for each spatially sorted (red closed circles) and shuffled (blue open circles) replicate (nine each); females **(a)** and males **(b)** are plotted separately. The dashed 1:1 line indicates when mean dispersal distances for pre- and post-invasion kernels are equal; points above the 1:1 line reflect an increase in mean dispersal distance from the beginning to the end of the experiment, while points below the 1:1 line reflect a decrease. In general, spatially shuffled replicates occur close to the 1:1 line, indicating little change in mean dispersal distance. Spatially sorted replicates are farther from the 1:1 line than shuffled replicates, suggesting evolutionary changes in dispersal distance that are consistent with our other findings (Fig. 3, Supplementary Figs. 4, 5, Supplementary Tables 4, 5).

Supplementary Tables

Supplementary Table 1 | Invasion extent model selection

Model	K	AIC	ΔAIC	AIC weight
~ $\beta_0 + \beta_{TRT} + \beta_{GEN} + \beta_{TRT\times GEN}$	7	1074.3	0.00	0.85
~ $\beta_0 + \beta_{TRT} + \beta_{GEN}$	6	1078.5	4.17	0.11
~ $\beta_0 + \beta_{GEN}$	5	1080.1	5.79	0.05
~ $\beta_0 + \beta_{TRT} + \beta_{GEN}^2$	6	1128.2	53.85	0.00
~ $\beta_0 + \beta_{GEN}^2$	5	1130.7	56.37	0.00
~ $\beta_0 + \beta_{TRT} + \beta_{GEN} + \beta_{TRT\times GEN}^2$	7	1131.7	57.41	0.00
~ $\beta_0 + \beta_{TRT}$	5	1162.7	88.34	0.00
~ β_0	4	1165.2	90.88	0.00

The parameters shown in this table are model intercept (β_0), effect of treatment (β_{TRT}), effect of generation (β_{GEN}), and the effect of the interaction between treatment and generation ($\beta_{TRT\times GEN}$).

Supplementary Table 2 | Invasion variance model selection

$\sigma_A{}^2{}_{TRT}$	$\sigma_W{}^2{}_{TRT}$	WAIC	ΔWAIC	WAIC weight
yes	no	929.9	0.00	0.50
yes	yes	929.9	0.01	0.50
no	no	943.4	13.42	0.00
no	yes	945.7	15.72	0.00

The columns $\sigma_W{}^2{}_{TRT}$ and $\sigma_A{}^2{}_{TRT}$ indicate whether the within- or among-replicate variances, respectively, were modeled as having a treatment effect (‘yes’) or not (‘no’). The fixed-effect structure of these models is represented by the top model in Supplementary Table 1.

Supplementary Table 3 | Model selection for additive genetic variance in dispersal

Sex	$V_A(\xi)$	WAIC	ΔWAIC	WAIC weight
Females	yes	2524.80	0.00	0.80
	no	2527.56	2.75	0.20
Males	yes	2388.26	0.00	0.65
	no	2389.45	1.19	0.36

The column $V_A(\xi)$ denotes whether or not the candidate model accounts for additive genetic (sire) variance in ξ, the mean of the PIG distribution (Methods). Model selection results are presented for females and males, which were analyzed separately.

Supplementary Table 4 | Common garden dispersal model selection (females)

Model	σ^2_{TRT}	WAIC	ΔWAIC	WAIC weight
~ $\beta_0 + \beta_{TRT} + \beta_{CGG}$	no	7688.3	0.00	0.15
~ $\beta_0 + \beta_{CGG}$	no	7688.5	0.21	0.14
~ $\beta_0 + \beta_{CGG} + \beta_{TRT\times CGG}$	no	7688.8	0.47	0.12
~ $\beta_0 + \beta_{CGG}$	yes	7688.9	0.57	0.12
~ $\beta_0 + \beta_{TRT} + \beta_{CGG} + \beta_{TRT\times CGG}$	no	7689.2	0.93	0.10
~ $\beta_0 + \beta_{TRT}$	no	7689.3	0.97	0.10
~ $\beta_0 + \beta_{TRT} + \beta_{CGG}$	yes	7689.5	1.24	0.08
~ $\beta_0 + \beta_{CGG} + \beta_{TRT\times CGG}$	yes	7689.6	1.26	0.08
~ $\beta_0 + \beta_{TRT} + \beta_{CGG} + \beta_{TRT\times CGG}$	yes	7690.8	2.54	0.04
~ $\beta_0 + \beta_{TRT\times CGG}$	no	7693.1	4.79	0.01
~ β_0	no	7693.2	4.89	0.01
~ $\beta_0 + \beta_{TRT} + \beta_{TRT\times CGG}$	no	7693.8	5.46	0.01
~ $\beta_0 + \beta_{TRT} + \beta_{TRT\times CGG}$	yes	7694.4	6.06	0.01
~ β_0	yes	7694.4	6.07	0.01
~ $\beta_0 + \beta_{TRT\times CGG}$	yes	7694.6	6.26	0.01
~ $\beta_0 + \beta_{TRT}$	yes	7694.6	6.33	0.01

The parameters shown in this table are model intercept (β_0), effect of treatment (β_{TRT}), effect of common garden generation (β_{CGG}), and the effect of the interaction between treatment and common garden generation ($\beta_{TRT\times CGG}$). We always modeled PIG mean (ξ_{jkl}) and shape (ω_{ijk}) parameters as having the same linear predictors (Methods), so for simplicity we only show one representative expression in this table. The column σ^2_{TRT} indicates whether the random-effect variances on kernel parameters were modeled as having a treatment effect ('yes') or not ('no').

WAIC support was broadly distributed among eight models, which comprised 90% of the cumulative WAIC weight. Collectively, these models provide support for an effect of treatment on the dispersal kernel. Of these eight models, seven included an effect of shuffle treatment on the kernel's mean (ξ) and shape (ω) and/or random effect variance. All models that contained an effect of treatment showed that female beetles descended from sorted invasion fronts had dispersal kernels with greater means (ξ) but similar shape parameters (ω) when compared to beetles descended from shuffled invasion fronts. Common garden generation had an effect on the mean and shape parameters, independent of treatment. In general, the dispersal kernel mean (ξ) decreased from the first to the second common garden generation, while ω increased. Thus, beetles descended from all invasions dispersed less far in the second common garden generation and had shorter-tailed dispersal kernels than in the first generation. Three of the eight top models include an interactive effect between the shuffle treatment and common garden generation. Under these models, females from the shuffle treatment experienced smaller parameter changes between the first and second common garden generations than their sorted counterparts (Supplementary Figure 4).

Supplementary Table 5 | Common garden dispersal model selection (males)

Model	σ^2_{TRT}	WAIC	ΔWAIC	WAIC weight
$\sim \beta_0 + \beta_{CGG}$	yes	8148.0	0.00	0.43
$\sim \beta_0 + \beta_{TRT} + \beta_{CGG}$	yes	8149.2	1.24	0.23
$\sim \beta_0 + \beta_{CGG} + \beta_{TRT\times CGG}$	yes	8149.5	1.56	0.20
$\sim \beta_0 + \beta_{TRT} + \beta_{CGG} + \beta_{TRT\times CGG}$	yes	8150.6	2.62	0.12
$\sim \beta_0 + \beta_{CGG}$	no	8154.6	6.66	0.02
$\sim \beta_0 + \beta_{TRT} + \beta_{CGG}$	no	8155.9	7.92	0.01
$\sim \beta_0 + \beta_{CGG} + \beta_{TRT\times CGG}$	no	8157.0	9.05	0.00
$\sim \beta_0 + \beta_{TRT} + \beta_{CGG} + \beta_{TRT\times CGG}$	no	8157.9	9.90	0.00
$\sim \beta_0 + \beta_{TRT\times CGG}$	yes	8162.5	14.49	0.00
$\sim \beta_0 + \beta_{TRT} + \beta_{TRT\times CGG}$	yes	8163.3	15.39	0.00
$\sim \beta_0 + \beta_{TRT}$	yes	8163.8	15.82	0.00
$\sim \beta_0$	yes	8164.1	16.11	0.00
$\sim \beta_0 + \beta_{TRT\times CGG}$	no	8169.2	21.27	0.00
$\sim \beta_0$	no	8169.6	21.68	0.00
$\sim \beta_0 + \beta_{TRT}$	no	8169.8	21.89	0.00
$\sim \beta_0 + \beta_{TRT} + \beta_{TRT\times CGG}$	no	8169.9	21.97	0.00

The parameters shown in this table are model intercept (β_0), effect of treatment (β_{TRT}), effect of common garden generation (β_{CGG}), and the effect of the interaction between treatment and common garden generation ($\beta_{TRT\times CGG}$). We always modeled PIG mean (ξ_{jkl}) and shape (ω_{ijk}) parameters as having the same linear predictors (Methods), so for simplicity we only show one representative expression in this table. The column σ^2_{TRT} indicates whether the random-effect variances on kernel parameters were modeled as having a treatment effect ('yes') or not ('no').

There were four top models comprising 97% of the cumulative WAIC weight. The single best model (43% WAIC weight) includes the fixed effect of common garden generation and treatment-specific variances in random effects. All four top models include treatment-specific variances in random effects, with greater among-population variance for sorted invasions; three of these models also include a fixed effect of treatment on kernel parameters. These parameter estimates suggest that males descended from spatially sorted invasions had a farther mean dispersal distance than males descended from shuffled invasions (Supplementary Figure 5). Parameter estimates show that males experienced a decrease in ξ from the first to second common garden generation, similar to what was observed in females (Supplementary Table 4). Shape parameters (ω) increased from the first to the second common garden generation – reducing kernel variance over time – again similar to the response observed in females (Supplementary Table 4).

Supplementary Table 6 | Pre-invasion dispersal kernel model selection

Sex	Model	σ^2_{TRT}	WAIC	ΔWAIC	WAIC weight
Females	~ β_0	no	1150.92	0.00	0.47
	~ β_0	yes	1151.86	0.95	0.29
	~ $\beta_0 + \beta_{TRT}$	yes	1153.63	2.71	0.12
	~ $\beta_0 + \beta_{TRT}$	no	1153.78	2.87	0.11
Males	~ β_0	yes	1279.34	0.00	0.38
	~ β_0	no	1279.53	0.20	0.35
	~ $\beta_0 + \beta_{TRT}$	no	1281.28	1.95	0.14
	~ $\beta_0 + \beta_{TRT}$	yes	1281.49	2.15	0.13

The parameters shown in this table are model intercept (β_0), and effect of treatment (β_{TRT}). We always modeled PIG mean (ξ_{jkl}) and shape (ω_{ijk}) parameters as having the same model structure (Methods), so for simplicity we only show one representative expression in this table. The column σ^2_{TRT} indicates whether the random-effect variances on kernel parameters were modeled as having a treatment effect (‘yes’) or not (‘no’). Model selection results are presented for females and males, which were analyzed independently. WAIC weights may not sum to 1 due to rounding.

Supplementary Table 7 | Common garden fecundity model selection

Model	*K*	AIC	ΔAIC	AIC weight
~ β_0	4	3402.7	0.00	0.52
~ $\beta_0 + \beta_{TRT}$	5	3404.7	2.00	0.19
~ $\beta_0 + \beta_{CGG}$	5	3404.7	2.00	0.19
~ $\beta_0 + \beta_{TRT} + \beta_{CGG}$	6	3406.7	4.00	0.07
~ $\beta_0 + \beta_{TRT} + \beta_{CGG} + \beta_{TRT \times CGG}$	7	3408.6	5.90	0.03

The parameters shown in this table are model intercept (β_0), effect of treatment (β_{TRT}), effect of common garden generation (β_{CGG}), and the effect of the interaction between treatment and common garden generation ($\beta_{TRT \times CGG}$). Fecundity was modeled as being negative-binomially distributed (Methods).

Supplementary Table 8 | Bottleneck size model selection

Model	K	AIC	ΔAIC	AIC weight
$\sim \beta_0$	1	500.09	0.00	0.52
$\sim \beta_0 + \beta_{TRT}$	2	502.00	1.91	0.20
$\sim \beta_0 + \beta_{CGG}$	2	502.13	2.05	0.19
$\sim \beta_0 + \beta_{TRT} + \beta_{CGG}$	3	504.07	3.98	0.07
$\sim \beta_0 + \beta_{TRT} + \beta_{CGG} + \beta_{TRT \times CGG}$	4	506.09	6.00	0.03

The parameters shown in this table are model intercept (β_0), effect of treatment (β_{TRT}), effect of common garden generation (β_{CGG}), and the effect of the interaction between treatment and common garden generation ($\beta_{TRT \times CGG}$). Bottleneck size was modeled as being Poisson distributed (Methods). The best-fitting model was the null model (52% AIC weight), suggesting that neither treatment nor generation had an effect on the size of the population bottleneck.
